# Suboptimal Bacteriological Quality of Household Water in Municipal Ibadan, Nigeria

**DOI:** 10.4269/ajtmh.23-0134

**Published:** 2024-01-02

**Authors:** Olumuyiwa S. Alabi, Ifeoluwa Akintayo, Jesutofunmi S. Odeyemi, Jeremiah J. Oloche, Chibuzor M. Babalola, Chukwuemeka Nwimo, Oluwafemi Popoola, Ondari D. Mogeni, Florian Marks, Iruka N. Okeke

**Affiliations:** ^1^ Department of Pharmaceutical Microbiology, Faculty of Pharmacy, University of Ibadan, Nigeria;; ^2^ Department of Pharmacology and Therapeutics, College of Health Sciences, Benue State University, Makurdi, Nigeria;; ^3^ Keck School of Medicine, Department of Population and Public Health Sciences, University of Southern California, Los Angeles, California;; ^4^ Department of Community Medicine, College of Medicine, University of Ibadan, Nigeria;; ^5^ Epidemiology, Public Health, Implementation & Clinical Development Unit, International Vaccine Institute, Seoul, South Korea;; ^6^ Cambridge Institute of Therapeutic Immunology and Infectious Disease, University of Cambridge School of Clinical Medicine, Cambridge Biomedical Campus, Cambridge, United Kingdom;; ^7^ Heidelberg Institute of Global Health, University of Heidelberg, Heidelberg, Germany;; ^8^ Madagascar Institute for Vaccine Research, University of Antananarivo, Antananarivo, Madagascar

## Abstract

Access to potable water is difficult for many African residents. This study evaluated the bacteriological quality of household water collected in the dry and wet seasons across five municipal local government areas (LGAs) in Ibadan, a large city in southwest Nigeria. A total of 447 water samples (dry season, *n =* 250; wet season, *n =* 197) were aseptically collected from a random sample of mapped households within Ibadan’s five municipal LGAs. The pH values and total aerobic and coliform bacterial counts were measured, and samples were screened for *Escherichia coli, Salmonella, Shigella*, and *Yersinia* by standard phenotypic techniques and multiplex polymerase chain reaction. The most common source of water was well (53.2%), followed by borehole (34%). None of the households used municipal tap water. Cumulatively, aerobic (*P* = 0.0002) and coliform (*P* = 0.0001) counts as well as pH values (*P* = 0.0002) changed significantly between seasons, with increasing and decreasing counts depending on the LGA. Nonpotable water samples were found to be very common during the dry (86.8%) and wet (74.1%) seasons. *Escherichia coli* spp., as indicators of recent fecal contamination, were isolated from 115 (25.7%) of the household water sources. Thirty three *Salmonella*, four enteroaggregative *E. coli*, and four enterotoxigenic *E. coli* isolates but no *Shigella* or *Yersinia* isolates were identified. This study revealed the absence of treated tap water and the poor quality of alternative sources with detectable pathogens in municipal Ibadan. Addressing the city-wide lack of access to potable water is an essential priority for preventing a high prevalence of feco-orally transmitted infections.

## INTRODUCTION

Water is essential for life, but a considerable number of households (or countries) in Africa still lack access to safe, potable water. Natural water sources are often contaminated either directly or indirectly by human and animal activities and hence require rigorous treatment before they can be declared potable.^1^ Urbanization offers the opportunity to centralize water treatment and dissemination, but where city waste management and water treatment facilities are sub optimal, as in most of Nigeria, water quality and shortage problems can be exacerbated. Municipal water and sanitation systems established in Nigerian cities during and shortly after colonial times fail to meet the need of the growing population such that substitute atmospheric, surface, and underground water resources are extensively exploited.^2^^–^^6^ Contamination from soil and sanitation shortfalls, as well as agricultural, industrial, and other environmental activities, contributes to the microbial load in groundwater.^7^^,^^8^ Notably, groundwater should have better microbial quality than surface water, because the coarse particulate network of the soil serves as a natural filter for microbial contaminants in both atmospheric and surface water percolating down the water table.^9^ However, the depth of the water and the porosity of the soil determine water purity, and wells and boreholes that are too close to underground sewers may be prone to contamination.^10^^,^^11^

According to the report of the WHO and UNICEF in 2017, about 4.5 billion people lacked access to safely managed sanitation as at the end of 2015 and 2.1 billion people in the world do not have readily available safe water at home.^12^ Among these 2.1 billion people, 844 million (40.2%) do not have a basic drinking water service and 159 million, 58% from African countries, depend on untreated water from surface water sources.^12^^,^^13^ About 60 million Nigerians are said to lack access to potable water, with 53% of the people living in rural areas and 28% of those in urban settlements believed to have no access to quality water sources.^14^

Ibadan is a large and densely populated city with over 2 million inhabitants but with no noticeable tap water supply. While the six outlying local government areas (LGAs) were largely excluded from water system planning, Ibadan’s five municipal LGAs were originally intended to receive tap-borne water from treatment plants just outside the center at Eleyele and Asejire plants. However, in spite of efforts to scale up supplies from Asejire, this plant was reportedly meeting less than 10% of the daily demand in 2004, when Ibadan’s population was roughly half of what it is today. Given this severe undercapacity and considerable population expansion in the interim, the vast majority of Ibadan residents resort to alternate sources of water either continuously or intermittently.^6^^,^^15^

The presence of pathogens in water for drinking or domestic purposes such as cooking, washing food items and utensils, personal hygiene, and even recreation is a serious public health threat if such contaminated water is ingested, particularly for children under 5 years old, the elderly, and the immunocompromised. Waterborne pathogens are not always detectable when present. Therefore, indicators of water contamination and, in particular, recent fecal contamination, are reliable means to determine the potability of water or to indicate significant transmission risks for enteric pathogens.^16^

Diarrheal disease is one of the most common consequences of consumption of enteric pathogen-contaminated water. According to the WHO,^17^ diarrheal disease has been reported to be the second leading cause of death among children less than 5 years old. In the year 2019, 370,000 children died as a result of diarrhea, and currently, yearly deaths of about 525,000 children less than 5 years old have been recorded.^17^ Nigeria accounts for the highest number of diarrheal disease deaths in Africa,^17^^,^^18^ the majority of which are preventable by providing safe drinking water, adequate sanitation, and good hygiene. To advocate for and potentially inform much needed efforts to improve the quality of existing domestic water sources, we evaluated and characterized the level of bacterial contamination of household water in municipal Ibadan during the dry and wet seasons.

## MATERIALS AND METHODS

### Study area.

The study was conducted in the five municipal LGAs of Ibadan, Oyo State, Nigeria. Ibadan is known to be the largest city in Nigeria, with approximately 2.6 million population as reported in the 2006 census and with a land surface area of about 828 km^2^ located within the coordinates 7.3775°N, 3.9470°E, West Africa. The five municipal LGAs of Ibadan, namely, Ibadan North (IBN), Ibadan South-West (IBSW), Ibadan North-East (IBNE), Ibadan North-West (IBNW), and Ibadan South-East (IBSE), have a land surface area of 128 km^2^ and a population of 1,338,659 at the year 2006 census.^19^

### Study design and sampling technique.

This was a descriptive cross-sectional study. Sample size calculations were as described by Kelsey et al.,^20^ with the aim to collect 250 water samples per season. A sampling frame was constructed using aerial maps to enumerate all structures in the purposively selected study area, as previously described by Pak et al.^19^ Abuja Geographic Information Systems software was used to randomly select sampling points (households) based on the sample size calculated within the entire sampling frame. The Global Positioning System (GPS) coordinates for the households involved in the study were taken with a GPS device and recorded accordingly, as previously described by Pak et al.^21^ The maps were generated using geographical coordinates of each of the water points on the ArcGIS Online basemap layer Environmental Systems Research Institute HERE, Garmin, Four square METI/NASA USGS (https://www.arcgis.com/home/index.html).

### Water sample collection and pH determination.

Field assistants were recruited and trained on how to aseptically collect water samples from the different sources in the mapped locations within the LGAs. Water samples were collected aseptically from what the interviewed head of household at the designated premises described as the major source of household water. The range of sources included wells (WW), boreholes (BHW), streams (SW), and water supplied by tanker vehicles and stored in tanks (TW). Samples were collected in the months of March and April 2018 (towards the end of the dry season) and again between October and November 2018 (at the end of the wet season). The water samples were collected aseptically into wide-mouth sterile glass bottles with tight screw cap closure.^16^ Samples from streams were collected at a point halfway between the edge and the center of the stream with the mouth of the bottle placed against the water current until it overflowed with the water; the bottle was then withdrawn and covered aseptically.^16^ Samples from open wells were collected by submerging the wide-mouth sterile bottle completely into the well, at the center point, with the aid of a strong sterile rope, and the bottle was then withdrawn and capped aseptically when nearly full.^16^ Samples from boreholes and water tanks were collected aseptically through the connected pipe outlets directly into the sterile bottles before closure. The water in the pipes was allowed to run for few seconds after disinfection of the mouth of the tap before collection into the bottles to eliminate external contaminants at the mouth of the taps.^16^ All the water samples were labeled after collection and transported to the laboratory in an icebox for analysis within 6 hours of collection.

The pH of the water samples was determined using an HI-2210 benchtop pH meter (Laboratory Analysis Ltd., Exeter, United Kingdom) according to the manufacturer’s specifications. Duplicate readings were taken, and the average pH value was recorded.

### Aerobic and coliform bacterial counts.

The water samples were analyzed for the presence of aerobic and coliform bacteria by the spread plate method on nutrient and MacConkey agar plates, respectively, as previously described by Koster et al.^22^ Briefly, after cold transportation to the laboratory and within 6 hours of collection, each water sample was 10-fold diluted with sterile distilled water. For each tested dilution, 100 µL was inoculated aseptically on nutrient agar (for aerobic counts) and MacConkey agar (for coliform counts) and spread using a sterile glass spreader over the agar surface. All dilutions were processed in duplicate, and the average colony-forming units per 1 mL (for aerobic counts) and per 100 mL (for coliform counts) of original sample were computed after incubation at 37°C for 24 hours. Samples with aerobic and coliform counts within the standard acceptable limits according to the Nigerian Industrial Standards Nigeria Standard of Drinking Water Quality (NSDWQ)^23^ and the WHO^24^ were noted and recorded.

### Isolation and identification of bacteria.

Water samples were analyzed for the presence of *Escherichia coli*, *Shigella* spp., *Yersinia* spp., and *Salmonella* spp. by using their respective enrichment and selective media. Briefly, 5 colonies showing typical *Escherichia coli* morphology on MacConkey plates used to compute coliform counts were streaked on eosin methylene blue (EMB) agar and incubated at 37°C for 18–24 hours. Cultures yielding colonies with a green metallic sheen typical of *E. coli* on the EMB agar plates were subcultured onto tryptone soy agar and then subjected to the indole test, hydrogen sulfide test, motility test, urease test, oxidase test, and citrate test. Isolates that were indole positive and hydrogen sulfide, urease, and oxidase negative were confirmed as *E. coli*. *Salmonella* spp. were isolated by filtering 500 mL of each water sample with a 0.22-µm membrane filter and culturing the filter in 10 mL of tryptic soy broth for 18–24 hours at 37°C. One hundred microliters of the 24-hour culture was preenriched in 5 mL of selenite-F broth and incubated for 24 hours at 37°C to facilitate the growth of the *Salmonella* spp. The selenite-F cultures were inoculated onto xylose lysine deoxycholate agar plates and incubated at 37°C for 18–24 hours in an inverted position. Black colonies on the plate were taken as presumptive *Salmonella* spp. The colonies were subjected to biochemical tests such as the indole, hydrogen sulfide, motility, urease, and citrate tests and polymerase chain reaction (PCR) for *invA* to confirm the identities of the presumptive *Salmonella* spp.^25^ *Yersinia* spp. were isolated by inoculating the membrane filter in 5 mL of phosphate-buffered saline for cold enrichment at –4°C for 21 days, after which it was then subcultured on yersinia selective agar and incubated at 35°C for 48 hours. Pure cultures of all the presumptive bacterial colonies were stored in Luria broth-glycerol (ratio, 50:50) at –80°C.

### Detection of diarrheagenic *E. coli* and confirmation of *Salmonella* spp.

Diarrheagenic *E. coli* was detected using a multiplex PCR protocol originally described by Aranda et al.,^26^ including modifications outlined by Odetoyin et al.^27^ Genomic DNA of the *E. coli* was obtained by boiling, and 1 µL of the extracted DNA was used as a template for amplification by PCR. The first multiplex PCR targeted enteroaggregative *E. coli* (EAEC) and enteropathogenic *E. coil* by using three primer pairs. A second multiplex PCR targeted Shiga toxin-producing *E. coli* and enterohemorrhagic *E. coil* by using two primer pairs, while the third PCR targeted enterotoxigenic *E. coli* (ETEC) and enteroinvasive *E. coli* in a three-primer pair multiplex reaction. Confirmation of the *Salmonella* spp. was by simplex PCR targeting the *invA* gene as previously described by Nucera et al.^25^ The PCR protocol was as follows: 5 minutes at 94°C prior to 35 cycles of 1 minute at 94°C, annealing temperature at 55°C for 1 minute, extension at 72°C for 1 minute, and a final extension of 10 minute at 72°C. The primers used and amplicon sizes are indicated in Supplemental Table 1.

### Statistical analysis.

Data generated were entered into the Statistical Package for the Social Sciences (SPSS) v. 23 (IBM, Inc., Chicago, IL) and expressed in percentage frequency distribution. The differences between the mean total aerobic and coliform bacterial counts for each water source during the dry and wet seasons were analyzed using the two-sample unequal variance Student’s *t* test for two-tailed distribution at a 95% CI. A *P* value less than 0.05 was considered statistically significant.

## RESULTS

### Sources and microbiological quality of water samples.

A total of 447 water samples including 250 and 197 water samples during the dry and wet seasons, respectively, were collected from households in the five municipal LGAs of Ibadan. Of the 250 water samples collected during the dry season, 131 (52.4%), 91 (36.4%), 26 (10.4%), and 2 (0.8%) were from wells, boreholes, water tanks, and streams, respectively, whereas 107 (54.3%), 61 (31.0%), 29 (14.7%), and 0 (0%) from those sources, respectively, were from the 197 water samples during the wet season (Table 1). The largest numbers of water samples were collected from IBNE during the dry (*n* = 61, 24.4%) and wet (*n* = 53, 26.9%) seasons, and most of these were from wells (*n* = 34, 55.7%, and *n* = 35, 66.0%, respectively). No water samples were collected from streams during the wet season or from 51 households that were initially sampled during the dry season because of nonaccessibility of the collection sites. This accounted for the lower number of water samples collected in the wet season (*n* = 197) than the dry season (*n* = 250). No household offered tap water as their source of household water.

**Table 1:** Distribution of household water samples collected from each local government area of municipal Ibadan in each season and analyzed in this study

Season and LGA	No. (%) of samples				
	Well	Borehole	Tank	Stream	Total
Dry season	*n =* 131	*n =* 91	*n =* 26	*n =* 2	*N =* 250
IBNE	34 (26.0)	15 (16.5)	11 (42.3)	1 (50.0)	61 (24.4)
IBNW	9 (6.9)	18 (19.8)	1 (3.8)	NS	28 (11.2)
IBN	25 (19.1)	25 (27.5)	6 (23.1)	1 (50.0)	57 (22.8)
IBSE	37 (28.2)	11 (12.1)	3 (11.5)	NS	51 (20.4)
IBSW	26 (19.8)	22 (24.2)	5 (19.2)	NS	53 (21.2)
Wet season	*n =* 107	*n =* 61	*n =* 29	*n =* 0	*N =* 197
IBNE	35 (32.7)	13 (21.3)	5 (17.2)	NS	53 (26.9)
IBNW	9 (8.4)	7 (11.5)	1 (3.4)	NS	17 (8.6)
IBN	16 (15.0)	18 (29.5)	13 (44.8)	NS	47 (23.9)
IBSE	27 (25.2)	8 (13.1)	3 (10.3)	NS	38 (19.3)
IBSW	20 (18.7)	15 (24.6)	7 (24.1)	NS	42 (21.3)

IBN *=* Ibadan North; IBNE = Ibadan North-East; IBNW = Ibadan North-West; IBSE = Ibadan South-East, IBSW = Ibadan South-West; LGA = local governmental area; NS = no sample.

**Figure 1.**
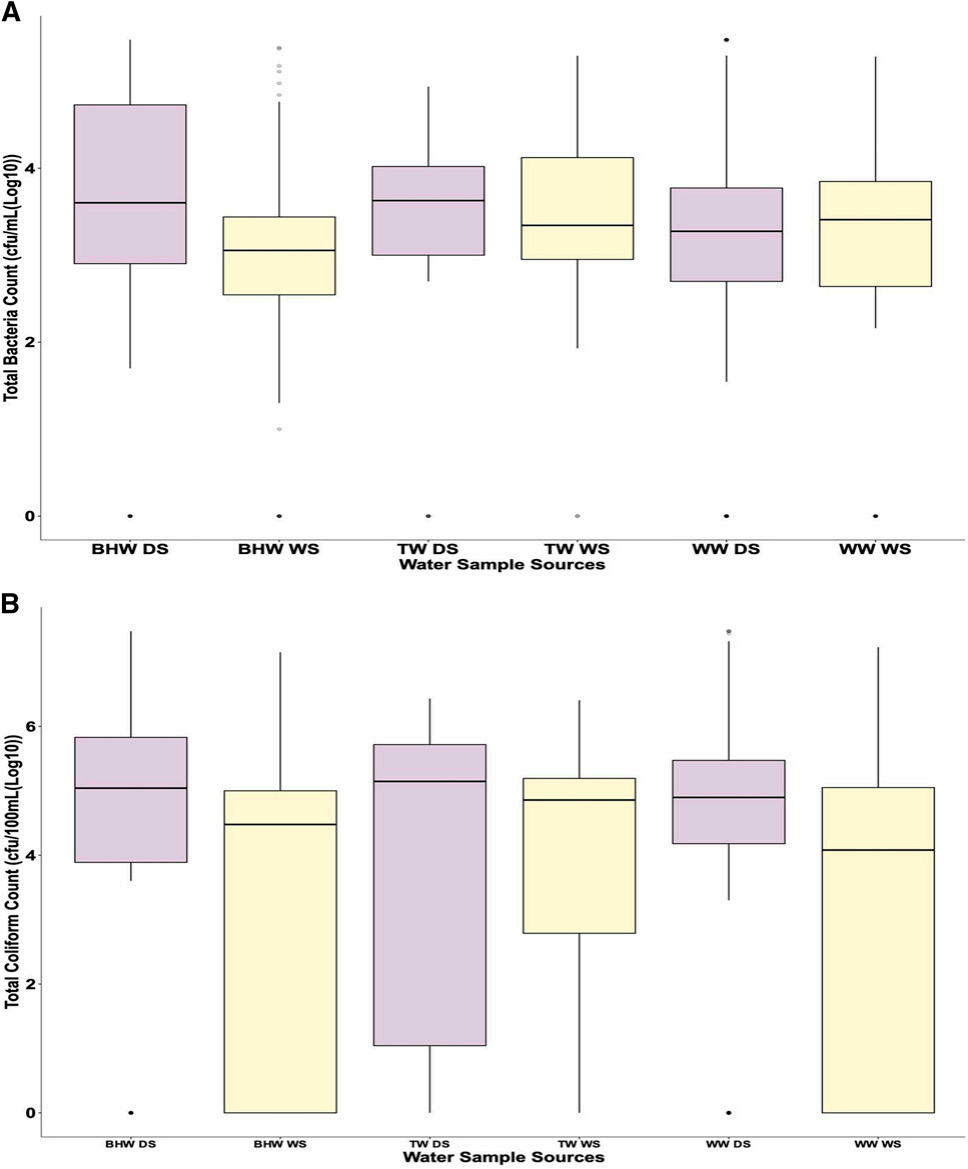
Seasonal variations of log_10_ mean total aerobic (**A**) and coliform (**B**) bacterial counts according to water sources. Each box represents the 25th and 75th percentiles, and the horizontal lines within the boxes represent median values. Whiskers above and below the boxes represent the highest and lowest microbial counts, respectively. BHW DS = borehole water, dry season; BHW WS = borehole water, wet season; TW DS = water stored in tank, dry season; TW WS = water stored in tank, wet season; WW DS = well water, dry season; WW WS = well water, wet season.

As shown in Figure 1, the maximum and median aerobic and coliform count values for water samples collected from most water sources in the LGAs during the dry season were higher than the values obtained during the wet season, except for water stored in tanks, for which the maximum aerobic count value was slightly higher during the wet than the dry season (Figure 1A and B). The coliform count values showed more variability between the 25% and 75% percentiles for all the water sources than the aerobic count values in both seasons, except for the coliform count values from well and borehole water samples during the dry season (Figure 1B). Water samples from streams were obtained only from IBNE and IBN LGAs during the dry season. The total aerobic and coliform counts for water samples from both wells and boreholes ranged from 0.0 to 3.0 × 10^5^ CFU/mL and 0.0 to 3.0 × 10^7^ CFU/100 mL, respectively, and the difference in the counts between the seasons was observed to be significant (*P* < 0.05). Water samples from tanks had counts within the range of 0.0–1.04 × 10^5^ CFU/mL and 0.0–1.04 × 10^7^ CFU/100 mL, with no significant difference in the aerobic (*P* = 0.2422) and coliform (*P* = 0.6518) counts, respectively, for both seasons (Table 2). The mean pH values for water samples from wells, boreholes, and water tanks were 6.42, 6.57, and 6.71, respectively, during the dry season and 6.22, 6.38, and 6.36, respectively, during the wet season. Seasonal variation observed in the pH values of water samples was significant for water from wells (*P* = 0.0086) but insignificant for water from boreholes (*P* = 0.0581) and water in tanks (*P* = 0.0601). Cumulatively, the seasonal variation in the pH values (*P* = 0.0002) and total aerobic (*P* = 0.0002) and coliform (*P* = 0.0001) counts for all the water samples combined were significant, as presented in Table 2.

**Table 2:** Bacterial counts and pH values of water samples during dry and wet seasons*

Water sources	Water quality parameters	Dry season					Wet season					*P* value†
		*N*	Mean	Median	Max	Min	*N*	Mean	Median	Max	Min	
Well	Aerobic count (×10^3^ CFU/mL)	131	28.42	2.25	300	0.0	107	10.19	1.76	290	0.0	0.0114
	Coliform count (×10^5^ CFU/100 mL)	131	18.05	1.0	300	0.0	107	4.80	0.26	209.5	0.0	0.0146
	pH value	131	6.42	6.44	8.01	5.22	107	6.22	6.31	8.14	5.38	0.0086
Borehole	Aerobic count (×10^3^ CFU/mL)	91	59.56	4.0	300	0.0	61	19.22	1.14	244	0.0	0.0021
	Coliform count (×10^5^ CFU/100 mL)	91	31.57	1.1	300	0.0	61	4.88	0.3	141.5	0.0	0.0036
	pH value	91	6.57	6.66	8.18	5.01	61	6.38	6.43	8.93	4.66	0.0581
Tank	Aerobic count (×10^3^ CFU/mL)	26	11.14	4.25	86.5	0.0	29	22.65	2.2	196	0.0	0.2422
	Coliform count (×10^5^ CFU/100 mL)	26	3.95	1.63	27	0.0	29	3.19	0.72	25.5	0.0	0.6518
	pH value	26	6.71	6.79	7.89	4.75	29	6.36	6.21	8.14	5.38	0.0601
Combined	Aerobic count (×10^3^ CFU/mL)	250	38.15	2.58	300	0.0	197	14.82	1.47	290	0.0	0.0002
	Coliform count (×10^5^ CFU/100 mL)	250	21.38	1.03	300	0.0	197	4.59	0.35	209.5	0.0	0.0001
	pH value	250	6.51	6.55	8.18	4.75	197	6.29	6.33	8.93	4.44	0.0002

CFU = colony-forming units; Max = maximum; Min = minimum.

* Arithmetic means were calculated.

† *P* value for comparison of difference in mean counts between dry and wet seasons; *P* < 0.05 is statistically significant.

**Figure 2.**
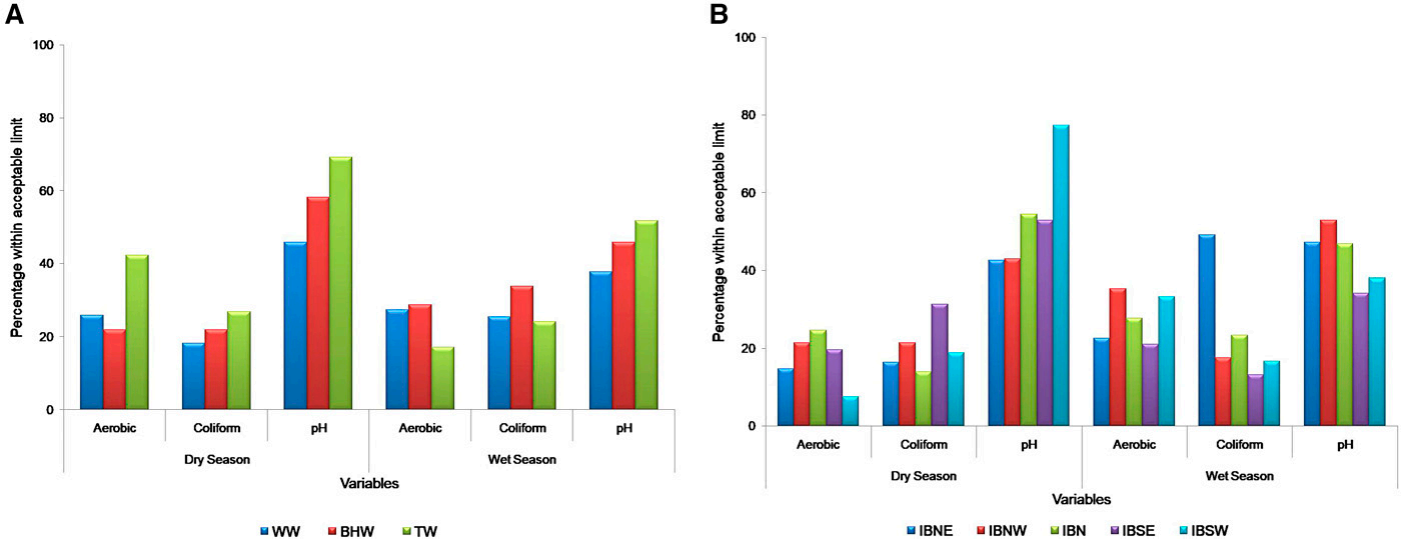
Percentage of water samples with aerobic and coliform counts and pH values within acceptable limits during the dry and wet seasons for each source of water (**A**) and each local government area (**B**). The WHO standard acceptable limit for aerobic count is 1.0 × 10^2^ CFU/mL, that for coliform count is 0 CFU/100 mL, and that for pH is 6.5 to 8.5. BHW = borehole water; IBN = Ibadan North; IBNE = Ibadan North-East; IBNW = Ibadan North-West; IBSE = Ibadan South-East; IBSW = Ibadan South-West; TW = water stored in tank; WW = well water.

Figure 2 shows the percentages of water samples with total aerobic and coliform counts and pH values that fall within the NSDWQ and WHO acceptable limits among the water samples collected during the dry and wet seasons according to the water sources and the LGAs. During the dry season, more water samples from water tanks had aerobic (42.3%) and coliform (26.9%) counts and pH values (69.2%) within acceptable limits than water samples from other sources like boreholes (aerobic count, 22.0%) and wells (coliform count, 18.3%, and pH, 45.8%) as shown in Figure 2A. However, during the wet season, more of the water samples collected in boreholes had aerobic and coliform counts within the acceptable limits (28.8% and 33.9%, respectively) than water samples from the other sources, with the lowest counts recorded for water tanks (17.2% and 24.1%, respectively). For the pH determination, water tanks had the highest percentage (69.2% and 51.7%, respectively) of water samples within the acceptable limit. Figure 2B shows the percentage of water samples with aerobic and coliform counts and pH values within acceptable limits in both seasons in the five municipal Ibadan LGAs. During the dry season, more of the water samples with aerobic and coliform counts and pH values within acceptable limits were recorded in IBN (24.6%), IBSE (31.4%), and IBSW (77.4%) LGAs, respectively, whereas the lowest percentages were recorded in IBSW (7.5%), IBN (14.0%), and IBNE (42.6%), respectively, compared with other LGAs. However, in comparison with other LGAs during the wet season, IBNW (35.3%) recorded the highest percentage of water samples with aerobic counts within the acceptable limit, IBNE (49.1%) recorded the highest for coliform counts, and IBNW (52.9%) recorded the highest for pH values (Figure 2B).

**Figure 3.**
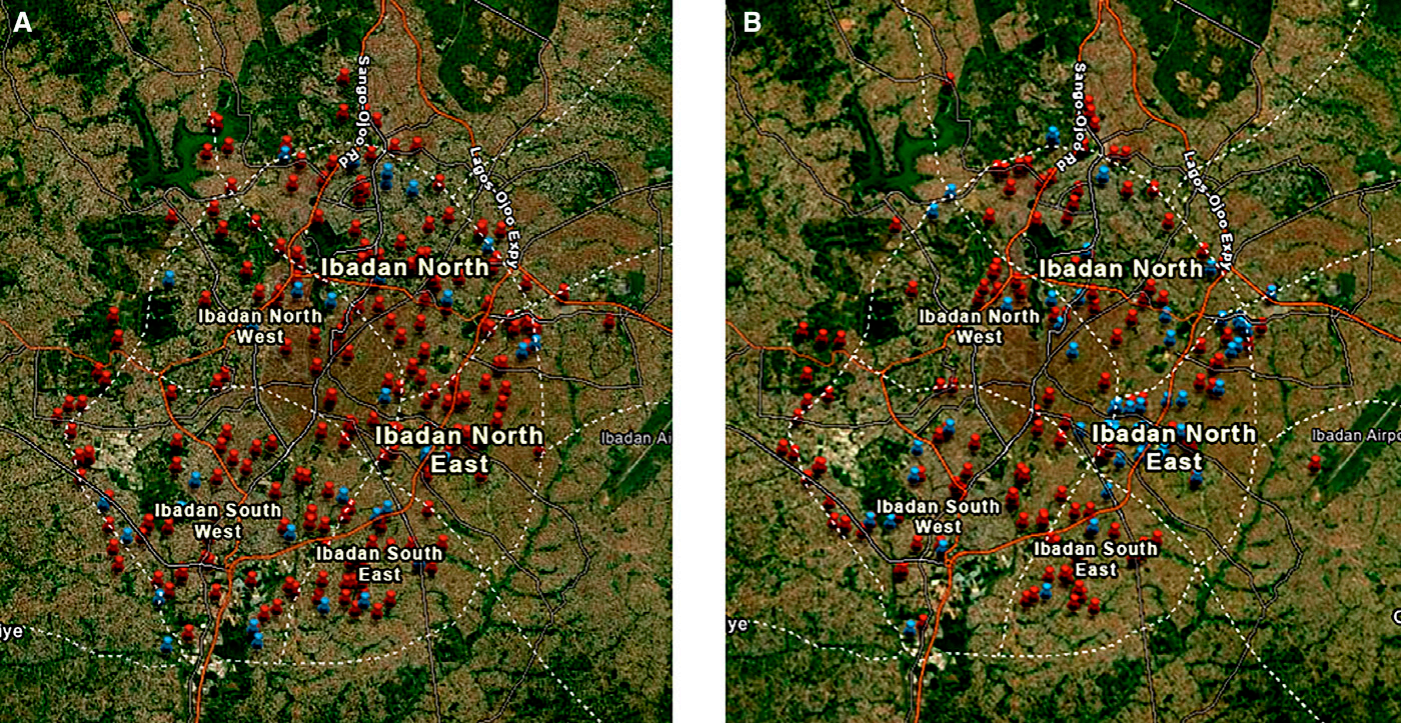
Map showing the locations of nonpotable and potable water sources within each local government area during the dry (**A**) and wet (**B**) seasons. 
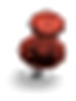
 = nonpotable water points; 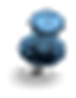 = potable water points.

Figure 3A and B are maps showing the spatial distribution of nonpotable and potable water sources across the different LGAs during the dry (Figure 3A) and wet (Figure 3B) seasons. From the maps, there are no clusters seen in both seasons, but both potable and nonpotable water sources were evenly distributed across the LGAs. During the dry season, 217 (86.8%) of the 250 water samples from the various sources in the five municipal LGAs were not potable, and this decreased to 146 of 197 (74.1%) water samples in the wet season. In almost all the LGAs in both seasons, especially during the dry season, more than 70% of the water samples obtained from each water source were nonpotable, as shown in Table 3.

**Table 3:** Percentage distribution of nonpotable and potable water samples across local government areas during dry and wet seasons

Season and LGA	No. (%) of samples							
	Well		Borehole		Tank		Combined sources	
	Potable	Nonpotable	Potable	Nonpotable	Potable	Nonpotable	Potable	Nonpotable
Dry season								
IBNE	4 (11.8)	30 (88.2)	1 (6.7)	14 (93.3)	1 (9.1)	10 (90.9)	6 (10.0)	54 (90.0)
IBNW	0 (0.0)	9 (100.0)	4 (22.2)	14 (77.8)	0 (0.0)	1 (100.0)	4 (14.3)	24 (85.7)
IBN	3 (12.0)	22 (88.0)	3 (12.0)	22 (88.0)	2 (33.3)	4 (66.7)	8 (14.3)	48 (85.7)
IBSE	5 (13.5)	32 (86.5)	2 (18.2)	9 (81.8)	2 (66.7)	1 (33.3)	9 (17.6)	42 (82.4)
IBSW	4 (15.4)	22 (84.6)	2 (9.1)	20 (90.9)	0 (0.0)	5 (100.0)	6 (11.3)	47 (88.7)
Wet season								
IBNE	18 (51.4)	17 (48.6)	8 (61.5)	5 (38.5)	0 (0.0)	5 (100.0)	26 (49.1)	27 (50.9)
IBNW	0 (0.0)	9 (100.0)	2 (28.6)	5 (71.4)	1 (100.0)	0 (0.0)	3 (17.6)	14 (82.4)
IBN	3 (18.8)	13 (81.2)	5 (27.8)	13 (72.2)	2 (15.4)	11 (84.6)	10 (21.3)	37 (78.7)
IBSE	3 (11.1)	24 (88.9)	1 (12.5)	7 (87.5)	1 (33.3)	2 (66.7)	5 (13.2)	33 (86.8)
IBSW	3 (15.0)	17 (85.0)	3 (20.0)	12 (80.0)	1 (14.3)	6 (85.7)	7 (16.7)	35 (83.3)

IBN = Ibadan North; IBNE = Ibadan North-East; IBNW = Ibadan North-West; IBSE = Ibadan South-East; IBSW = Ibadan South-West; LGA = local governmental area.

### Fecal indicator and pathogen detection.

**Figure 4.**
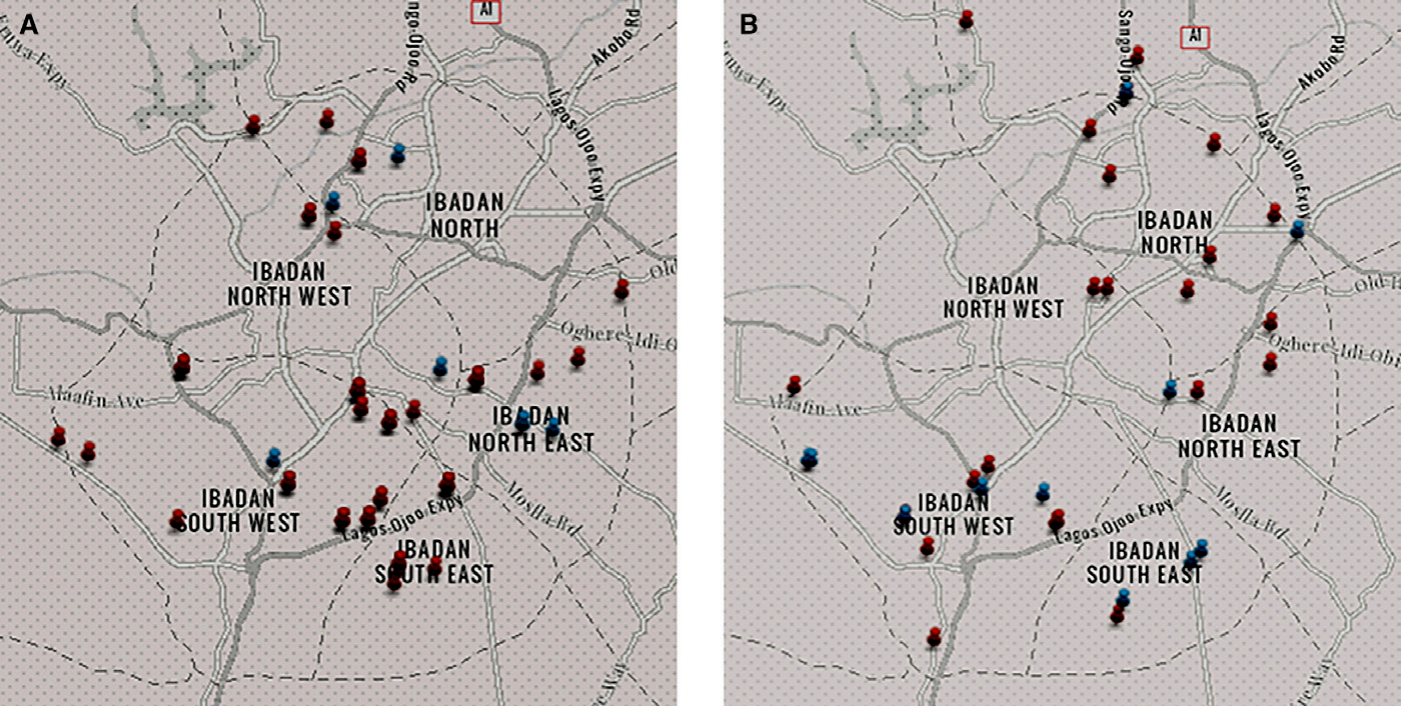
Spatial distribution of water sources contaminated with *Escherichia coli* and *Salmonella* spp. during the dry (**A**) and wet (**B**) seasons. 
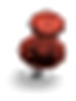
 = *Escherichia coli*; 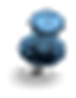 = *Salmonella* spp.

A total of 115 *E. coli* isolates were found, 76 (66.1%) and 39 (33.9%) during the dry and wet seasons, respectively. There were 33 *Salmonella* isolates, 16 (48.5%) and 17 (51.5%) during the dry and wet seasons, respectively (Table 4). No *Shigella* spp. or *Yersinia* spp. were isolated from any of the water sources in either season. *Escherichia coli* isolates were commonly recovered from underground water sources, with 57 (75%) and 17 (22.4%) isolates from wells and boreholes during the dry season and 29 (74.4%) and 5 (12.8%), respectively, during the wet season. Similarly, *Salmonella* spp. were isolated more often from wells than from other sources during the dry (*n* = 9, 56.3%) and wet (*n* = 12, 70.6%) seasons, as well as from water tanks (*n* = 6, 37.5%) during the dry season (Table 4). The distribution of water sources contaminated with *E. coli* and *Salmonella* spp. among the LGAs is shown on maps in Figure 4. Eight diarrheagenic *E. coli* (DEC) strains were identified among the *E. coli* isolates. Four of the DEC strains positive for the CVD432 locus, and therefore identified as EAEC, were isolated from same well water point (7.370213°N, 3.919472°E) during the dry season in IBNE LGA. The other four DEC strains harboring the *elt* gene and identified as ETEC were isolated from wells and water tanks in IBNW (*n* = 2, 50%), IBN (*n* = 1, 25%), and IBSE (*n* = 2, 25%) LGAs during the wet season (Figure 4). As shown in Figure 4, there was no clustering of locations from which fecally derived organisms or enteric pathogens were isolated.

**Table 4:** Percentage distribution of the isolated *Escherichia coli* and *Salmonella* spp. in relation to the sources, LGAs, and seasons

Season and LGA	No. (%) of samples							
	Well		Borehole		Tank		Combined	
	*E. coli*	*Salmonella*	*E. coli*	*Salmonella*	*E. coli*	*Salmonella*	*E. coli*	*Salmonella*
Dry season	*n =* 57	*n =* 9	*n =* 17	*n =* 1	*n =* 2	*n =* 6	*N =* 76	*N =* 16
IBNE	16 (28.1)	4 (44.4)	3 (17.6)	0 (0.0)	1 (50.0)	6 (100)	20 (26.3)	10 (62.5)
IBNW	0 (0.0)	0 (0.0)	1 (5.9)	1 (100)	0 (0.0)	0 (0.0)	1 (1.3)	1 (6.3)
IBN	4 (7.0)	3 (33.3)	7 (41.2)	0 (0.0)	0 (0.0)	0 (0.0)	11 (14.5)	3 (18.8)
IBSE	20 (35.1)	0 (0.0)	0 (0.0)	0 (0.0)	0 (0.0)	0 (0.0)	20 (26.3)	0 (0.0)
IBSW	17 (29.8)	2 (22.2)	6 (35.3)	0 (0.0)	1 (50.0)	0 (0.0)	24 (31.6)	2 (12.5)
Wet season	*n =* 29	*n =* 12	*n =* 5	*n =* 4	*n =* 5	*n =* 1	*N =* 39	*N =* 17
IBNE	5 (17.2)	1 (8.3)	0 (0.0)	2 (50.0)	0 (0.0)	0 (0.0)	5 (12.8)	3 (17.6)
IBNW	5 (17.2)	0 (0.0)	1 (20.0)	1 (25.0)	0 (0.0)	0 (0.0)	6 (15.4)	1 (5.9)
IBN	9 (31.0)	0 (0.0)	1 (20.0)	0 (0.0)	4 (80.0)	0 (0.0)	14 (35.9)	0 (0.0)
IBSE	8 (27.6)	6 (50.0)	0 (0.0)	1 (25.0)	1 (20.0)	1 (100)	9 (23.1)	8 (47.1)
IBSW	2 (6.9)	5 (41.7)	3 (60.0)	0 (0.0)	0 (0.0)	0 (0.0)	5 (12.8)	5 (29.4)

IBN = Ibadan North; IBNE = Ibadan North-East; IBNW = Ibadan North-West; IBSE = Ibadan South-East; IBSW = Ibadan South-West; LGA = local governmental area.

## DISCUSSION

Clean, potable water is essential for public health and in large cities is most reliably piped from treatment plants.^9^^,^^28^ This study has shown the complete absence of piped water in households in municipal Ibadan city and hence widespread dependence on alternate, mostly groundwater, sources for domestic purposes. Ibadan has struggled, largely unsuccessfully, to provide pipe-borne water to residents in its center for over three-quarters of a century.^3^^,^^6^^,^^15^ The difficulty in obtaining pipe-borne water in the city and the consequent reliance on streams contaminated with solid waste with a near absence of safe sanitation accounted for its mid-20th century devastation by cholera and other waterborne diseases.^6^ As a result, residents have resorted to individual and communally owned wells and boreholes as sources of household water in this dense municipality, which arises from decades of failure of the municipal water supply and has profound implications for the transmission of infectious pathogens.

Although the WHO recommended that coliform counts should be determined in cells per 100 mL of water sample using the membrane filtration method, the spread plate method was used to derive both aerobic and coliform counts in this study. The rationale behind this decision was consequent to the results of a previous pilot study^52^ carried out at a location closer to the study area, where plates with colonies too numerous to count were recovered using the membrane filtration method. Our experience with the pilot study, coupled with resource limitations which did not allow us to process samples by both spread plate and membrane filtration, therefore made it necessary to conduct this main study by using methods that would feasibly yield countable colonies for most samples. The high counts seen in a majority of samples in this study demonstrate that membrane filtration would not have yielded counts for most samples.

Total aerobic bacterial and coliform counts from household water samples evaluated in this study ranged from 0.0 to 3.0 × 10^5^ CFU/mL and 0.0 to 1.92 × 10^7^ CFU/100 mL, respectively, during the dry season and 0.0–3.0 × 10^5^ CFU/mL and 0.0–2.095 × 10^7^ CFU/100 mL, respectively, during the wet season. The counts were, for most of the samples, above the standard limit stated in the NSDWQ^23^ and WHO^24^ water quality guidelines. Our report follows the historical record and previous independent studies on the microbiological quality of household water in Ibadan that also reported the poor quality of household water sources in the metropolis of Ibadan. Odeleye and Idowu^29^ investigated the levels of total coliforms and fecal coliforms in hand-dug wells and boreholes in five LGAs in the municipal city of Ibadan. From their report, 96% of the hand-dug wells were highly contaminated, with total coliform and fecal coliform counts ranging between 0 and 160 × 10^3^ CFU/100 mL and 0 and 22 × 10^3^ CFU/100 mL, respectively. Ayantobo et al.^7^ evaluated the quality of 101 hand-dug wells in four of the LGAs covered in our study. Coliform and *E. coli* counts as high as 685 CFU/100 mL and 74.09 CFU/100 mL, respectively, were reported, and many of the wells were unprotected. Oloruntoba and Sridhar^3^ evaluated the bacteriological quality of water from wells, springs, boreholes, taps, and water stored in containers within the urban setting of Ibadan during the dry and wet seasons. Clearly, transfer of the responsibility for urban water sourcing from the state government to the household level has placed the majority of Ibadan residents at risk for waterborne diseases.^6^ In particular, high coliform counts were recorded among the water samples from wells. Wells were the most common household water source we encountered and also the most contaminated among the water sources.

The dire picture our study painted for metropolitan Ibadan applies to the outskirts of the city^30^ and other parts of southwest Nigeria,^31^^,^^32^ as well as similar settings elsewhere in Nigeria^33^^–^^35^ and Africa.^36^^,^^37^ For example, vended drinks produced with contaminated groundwater were implicated in the massive 2015 typhoid outbreak in Kampala, Uganda. However, in spite of the problem’s intransigence, and the population health risk that microbiologically unsafe water poses, water quality improvements are underprioritized. Awareness of the problem is limited, as much-needed water quality surveys are uncommon in Nigeria and similar settings and are typically performed only in response to devastating outbreaks.^38^ Idowu et al.^32^ reported high levels of contamination in more than 70% of water samples collected from both protected and unprotected wells in Sagamu, located in Ogun State, southwestern Nigeria. In another study carried out in Port Harcourt, in southern Nigeria, Kumpel et al.^8^ reported poor quality of water especially during the rainy season. Amold et al.^39^ in Ghana made a similar report, where 87% of evaluated hand-dug wells were contaminated with bacteria. Installation of wells or boreholes close to sanitary facilities, waste dumps, industrial effluent discharge areas, and burial grounds greatly contributed to microbial pollution of water and is almost impossible to avert because their digging is unplanned.^7^^,^^8^ Groundwater quality is further compromised when contaminated either directly or indirectly by humans and animals. Often, communal fetchers use ropes and containers to draw water in these largely hand-drawn wells. These, alongside other factors, can account for the high aerobic and coliform counts observed in the well and borehole water samples in this study. In the case of water stored in tanks, microbial contamination may arise from the supply source but could also be attributed to household-level contamination. Oloruntoba and Sridhar^3^ observed that at the household level, water quality significantly deteriorated after collection and storage because of poor handling.

Contamination of water sources, particularly groundwater, is often at its highest during the wet season rather than the dry season,^8^^,^^30^^,^^40^^,^^41^ but the reverse was seen in some studies. Akinyemi et al.^42^ reported a pronounced intensity of insecurity in household water during the dry season compared with that in the rainy season in a recent panel study designed to examine the effect of seasonal variations on household water security in an urban community in southwest Nigeria. Rabiu et al.,^43^ in a study on the microbiological quality of water from Watari Dam, Kano State, Nigeria, reported higher bacterial counts during the dry season than the wet season, but in a recent study carried out in Ibadan, more pathogens were recorded during the wet season.^30^ Akrong et al.,^44^ in a study of drinking water sources (such as streams, lakes, and boreholes) in communities surrounding Lake Bosomtwe in the Ashanti region of Ghana, reported significantly higher bacterial counts during the dry season than the wet season. Outside Africa, Atherholt et al.^45^ studied seasonal patterns of coliform bacteria in domestic wells in New Jersey by examining a statewide database generated during real estate transactions involving 10 years of water quality data from 93,447 samples obtained from 78,207 wells. They reported higher proportions of coliform bacteria in wells during the warm weather months. In this study, higher aerobic and coliform counts were observed during the dry season than during the wet season, especially among the water samples collected from wells and boreholes, and the differences in the aerobic and coliform counts in the water samples during the dry and wet seasons were significant (*P* < 0.05) in some of the LGAs. Although the higher counts recorded during the dry season rather than the wet season in some LGAs in this study seem to contradict the common belief that rain water runoff commonly increases the level of contaminants in water sources, especially when the rain is heavy, the possibility that the variation in counts is influenced mostly by spatial rather than seasonal factors is a possibility.^24^

Most bacteria, including human pathogens, are neutrophiles, that is, they grow optimally around neutral pH, usually between pH 6.5 and 7.5.^46^ The mean dry season pH of 6.51 recorded in this study falls within that range, while the mean wet season pH of 6.29 is slightly lower. This suggests pH-facilitated bacterial growth in the dry season that might be slightly tempered during the wet season, as evidenced by the relatively lower aerobic and coliform counts we recorded during the wet season. Although significant changes in mean total pH values for both seasons were recorded in this study, based on the variations observed in pH values in relation to the aerobic and coliform counts in water samples from either wells, boreholes, or water tanks in some of the LGAs, the significant change observed may not be the result of a seasonal influence but possibly a spatial one. It is important to note that metabolic activities of microorganisms, particular those contaminated with organic matter, generate several metabolites that can alter the pH of the environment,^46^ and therefore, it can also be suggested that the pH changes observed in this study may be influenced by spatial rather than seasonal factors.

The presence of *E. coli* marks water as high risk for containing enteric pathogens, which are much less likely to be cultured when present.^16^^,^^47^ In this study, *E. coli* strains were isolated predominantly from wells and boreholes, but not from streams, during both seasons. This strongly suggests that fecal contamination of groundwater and the higher recovery from wells, which are in general shallower than boreholes, support this hypothesis. *Escherichia coli* was most commonly recovered from wells in IBNE, IBSE, and IBSW LGAs in the dry season and from IBN in the wet season.

*Salmonella* spp. are only infrequently isolated from water when present, typically after enrichment. Therefore, the high recovery rates found in this study were very concerning. From wells in IBNE, *Salmonella* isolates were most commonly recovered in the dry season, but in IBSW, we isolated *Salmonella* more commonly in the rainy season. In IBNW LGA, one isolate each was recovered from borehole water from both seasons, but in IBN, recovery was made in water samples from wells only during the dry season. Notably, in IBSE, *Salmonella* was recovered from wells, boreholes, and watertanks during the wet season only. Although the serovars of the isolated *Salmonella* spp. were not reported in this study, they will be reported in our subsequent study. The variations in recovery of *E. coli* and *Salmonella* isolates from the water samples in the different LGAs in this study suggest that *Salmonella* may be present year-round. Moreover, there was no geographic clustering of fecally contaminated water and/or water samples from which enteric pathogens were isolated. The city-wide, nonseasonal recovery of these bacteria appears to be an indicator of diarrhea risk,^48^ which should be mitigated by more general interventions. The influence of environmental and human*/*animal factors on microbial contamination of water sources was not considered in this study. However, previous studies in Ibadan have identified most of these factors (sinking of wells/boreholes close to sanitary facilities, waste dumps, agricultural/industrial effluent discharge areas, and burial grounds, etc.) as contributing significantly to household water contamination.^3^^,^^7^^,^^15^

Diarrheagenic *E. coli* and *Salmonella* contribute significantly to the global diarrheal disease burden, especially in low-income countries.^17^^,^^49^ It has recently been determined that more than 60% of children under 5 years of age in northern Ibadan carry DEC and/or *Salmonella*, and the data suggest that risk of infection is similarly high in other parts of the city, where typhoid and invasive salmonellosis are also very common.^50^^–^^52^ Although other waterborne pathogens were either not recovered or not looked for, in this study, the high counts of total aerobic bacteria and coliform bacteria and the common recovery of fecal indicator *E. coli* and related pathogens suggest that other pathogens are likely present and that all but a few household water sources in municipal Ibadan are unsafe.

Vaccines that can protect against some waterborne pathogens exist,^53^^,^^54^ but these are unavailable or poorly deployed in Ibadan. The risk for residents, particularly the most vulnerable, i.e., the very young and the very old, is considerable, and interventions to halt the spread of these pathogens need to be urgently implemented.

## CONCLUSION

In conclusion, this study found that most household water sources in municipal Ibadan were highly contaminated by bacteria, particularly during the dry season. Water from these sources is unfit for drinking and other household purposes that could allow the ingestion of pathogens. Frequent detection of *E. coli* substantiates that most household water supplies are fecally contaminated. The recovery of overt pathogens, *Salmonella* spp. and EAEC and ETEC strains, points to these water sources as prime hazards. Ibadan requires citywide improvement in water, and likely sanitation, and until this is achieved, it is important to ensure that residents properly treat water prior to domestic use.

## Supplemental files

10.4269/ajtmh.23-0134 Supplemental Materials
