# Supplementary material for: Suboptimal Bacteriological Quality of Household Water in Municipal Ibadan, Nigeria
Source: Am J Trop Med Hyg. 2024 Jan 2;110(2):346–55. doi: 10.4269/ajtmh.23-0134 (PMC10859799; doi:10.4269/ajtmh.23-0134)
Supplement: Supplemental Materials [file tpmd230134.SD1.pdf]

TABLE S1

Primers for detection of *E. coli* pathotypes and *Salmonella* spp. confirmation

Target Strain	Target Genes		Primer Sequence	Band sizes	References
EAEC	CVD432	F	CTGGCGAAAGACYTGTATCAT	630	[26]
		R	CAATGTATAGAAATCCGCTGTT		
EPEC	*Eae*	F	CTGAACGGCGATTACGCGAA	917	[26]
		R	CCAGACVGATACGATCCAG		
EPEC	*bfpA*	F	AATGGTGCTTGCGCTTGCTGC	326	[26]
		R	GCCGCTTTATCCAACCTGGTA		
EHEC/STEC	*stx1*	F	ATAAATCGCCATTCGTTGACTAC	255	[26]
		R	AGAACGCCCACTGAGATCATC		
	stx2	F	GGCATGTCTGAAACTGCTCC	180	[26]
		R	TCGCCAGTTATCTGACATTCTG		
ETEC	*Lt*	F	GGCGACAGATTATACCGTGC	450	[26]
		R	CGGTCTCTATATT CCCTGTT		
	St	F	ATTTTTMTTTCTGTATTRTCTT	190	[26]
		R	CACCCGGTACARGCAGGATT		
EIEC	*ipaH*	F	GTTCCTTGACCGCCTTTCCGATACCGTC	600	[26]
		R	GCCGGTCAGCCACCCTCTGAGAGTAC		
InvA SAL	*invA*	F	GTGAAATTATCGCCACGTTCGGGCAA	187	[25]
		R	TCATCGCACCGTCAAAGGAACC		
